# Morphologic Evaluation of Dentoalveolar Structures after Corticotomy-Assisted Orthodontic Treatment in Romanian Adult Patients

**DOI:** 10.3390/medicina58040468

**Published:** 2022-03-23

**Authors:** Irinel Panainte, Dorin-Horațiu Nenovici, Marius Mariș, Dan-Cosmin Șerbănoiu, Claudiu Vartolomei, Mariana Păcurar

**Affiliations:** 1 Department of Orthodontics, Faculty of Dentistry, George Emil Palade Univeristy of Medicine and Pharmacy, 540142 Târgu Mureș, Romania; irinel.panainte@yahoo.com (I.P.); dorin.nenovici@gmail.com (D.-H.N.); serbanoiu.dancosmin@gmail.com (D.-C.Ș.); claudiu.vartolomei@gmail.com (C.V.); 2 Department of Orthodontics, Faculty of Dentistry, Titu Maiorescu University, 004051 Bucharest, Romania; marius@drmaris.ro

**Keywords:** orthodontics, corticotomy, bone thickness, tooth movement, root resorption, root length, cortical bone

## Abstract

*Background and Objectives*: Corticotomy-facilitated orthodontics is an approach that can be useful in treating complex orthodontic cases and that could enhance the rate of tooth movement. The aim of this study was to evaluate the changes that occurred in the buccal cortical bone and at the root level after an orthodontic treatment when corticotomy was used, in Romanian patients. *Materials and Methods*: After dividing the subjects into two groups (maxillary and mandibular corticotomy), based on CBCT, measurements were made of the thickness of the cortical buccal bone at the cervical, median and apical level, and of the root length at T0 (before corticotomy) and T1 (6 months after surgery). Several tests were used for statistical analysis of the data. *Results*: In the maxillary arch, the bone thickness measured after corticotomy in males was 0.64 mm at the cervical level, 0.53 mm at the medial level and 0.30 mm in the apical area. In females, the values were 0.46 mm (cervical), 0.37 mm (medial) and 0.36 mm (apical). In the lower arch, the values obtained for these three regions were 0.37 mm, 0.30 mm and 0.37 mm for males and 0.58 mm, 0.32 mm and 0.43 mm for female subjects. All values were statistically significant. The root length for the lower teeth at T0 was 11.98 ± 2.24 mm at T0 and 11.97 ± 2.24 mm at T1. For the upper teeth, the root length at T0 was 13.83 ± 2.28 mm and 13.81 ± 2.28 mm. *Conclusions*: Comparing the measurements, it was observed that the biggest changes in the cortical bone were at the cervical level. In the maxillary arch, the most significant modifications were registered at the canines and the level of the first premolars, and in the lower arch at the incisors level. The measured root resorption of the teeth was considered to be statistically insignificant.

## 1. Introduction

The orthodontic movement of the teeth is considered to be a periodontal phenomenon because, during orthodontic treatment, some changes occur in the supporting structures of the tooth, such as the compression of the periodontal ligament, resorption and bone apposition [1]. Although the prevalence of malocclusions is quite high in the general population, patients still refuse to visit an orthodontic specialist in order to improve their status, the main reason being the prolonged duration of the treatment [2]. It is well known that a prolonged duration of treatment can lead to side effects such as demineralization of the enamel, cavities, periodontal inflammation or even root resorption [3].

This is one of the main reasons why various procedures have been used to try to enhance the pace of tooth movement over time. Kale et al. observed that the rate of tooth displacement may vary depending on the local or systemic application of some drugs [4]; a similar observation was made by Tyrovola et al. in their study conducted in the same direction [5]. In order to obtain a faster movement of the teeth but also to eliminate the possible negative effects on the dental and periodontal support, a technique adjuvant to classical orthodontic therapy has recently been proposed—corticotomy [1]. Originally proposed by Kole, he suggested that performing osteotomies on the alveolar process would facilitate the movement of orthodontic teeth, as the bone cortex loses its rigidity [6]. Further studies of this technique concluded that the facilitation of tooth movement is actually achieved by an acceleration in local cellular metabolism, known as the Regionally Acceleratory Phenomenon (RAP). The changes involve a reduction in bone density, while the volume of the bone matrix remains unchanged [7,8].

Observing this phenomenon, Wilcko et al. proposed a new surgical technique—Periodontally Accelerated Osteogenic Orthodontics (PAOO) [9]. The authors hypothesized that osteopenia following surgical trauma reduces the resistance of bone to tooth displacement, thereby accelerating the degree of tooth movement during orthodontic therapy. Therefore, it has been found that PAOO creates a larger space for buccal root movement during orthodontic expansion, with minimal root resorption and bone dehiscence compared with the tooth displacement obtained with conventional orthodontic therapy [10,11].

In the literature there are currently a number of clinical studies [12,13] that highlight the potential benefits of combining corticotomy with orthodontic therapy, and that demonstrate the changes that occur in the dento-periodontal structures. Among the indications of PAOO are shortening the period of treatment and solving more challenging clinical situations, such as traction on the arch of the impacted canines; closing the old post-extraction spaces; obtaining a slight expansion; resolving the open bite; and molar intrusion. It is also expected that post-treatment stability will improve. In a series of experimental animal studies [14,15], it was observed that the rate of tooth displacement increases, with some bone structural changes. Despite this evidence, which proves that some benefits could be obtained with this approach, there is still reluctance among both patients and specialists when it comes to including this approach in a treatment plan.

Therefore, the purpose of this retrospective study was to evaluate the changes in the buccal cortical bone at the cervical, median and apical levels, as well as the changes that occurred in the root length following orthodontic treatment when using corticotomy in Romanian adult patients.

## 2. Materials and Methods

### 2.1. Study Design

The study consisted of 60 patients (28 men and 32 women) aged between 20 and 40 (men) and 24 and 46 (women), who received orthodontic treatment in a private clinic. The study was conducted in accordance with the Declaration of Helsinki and was approved by the Ethics Committee of George Emil Palade University of Medicine and Pharmacy (27 January 2022). Written informed consent was procured from every patient after the study design and protocol were explained. The sample was set up in such a way as to use each patient as his own control, thereby increasing the power of the small sample.

### 2.2. Inclusion Criteria

- Moderate to severe dental crowding;
- Class 1 skeletal pattern;
- Normal growth pattern;
- Angle’s Class I or Class II molar relationship;
- No extractions or congenitally missing teeth;
- No periodontal problems (gingival recession or root/bone resorption);
- Approximately the same duration of treatment time.

### 2.3. Exclusion Criteria

- Severe craniofacial abnormalities;
- Patients under anti-inflammatory medication;
- Systemic diseases (genetic, metabolic and endocrine diseases, inflammatory diseases).

### 2.4. Orthodontic Treatment

The case of each patient included in the study was documented with panoramic radiograph, lateral cephalogram, study models and intraoral and extraoral photographs. All patients had orthodontic treatment with a 0.22 inch slot, passive self-ligating braces, Damon prescription (Ormco BV) fully bonded to the second molars. Brackets were applied using the self-etching technique using Transbond XT material (3M, Unitek, Monrovia, CA, USA), due to its hybrid filling and high resistance to compression and bending. After the alignment of the teeth was completed (a heat-activated archwire of 0.18 CuNiTI was in place-T0), the subjects underwent cone beam computer tomography (CBCT).

### 2.5. Surgical Phase

**Figure 1:**
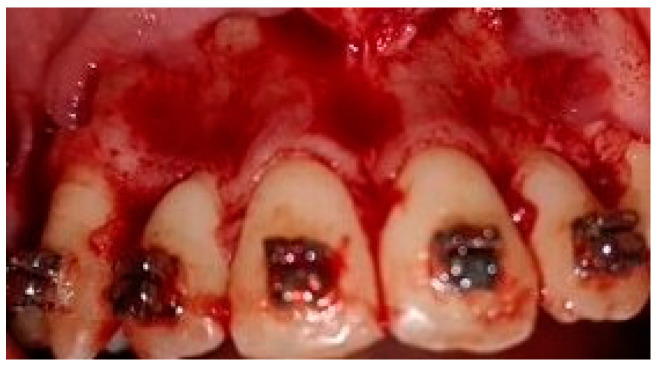
Full thickness flap before corticotomy.

**Figure 2:**
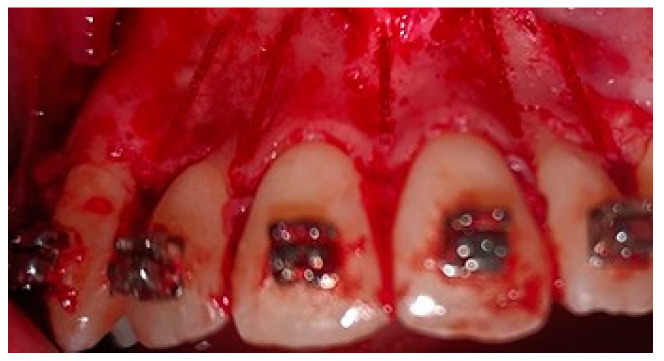
Corticotomy lines.

**Figure 3:**
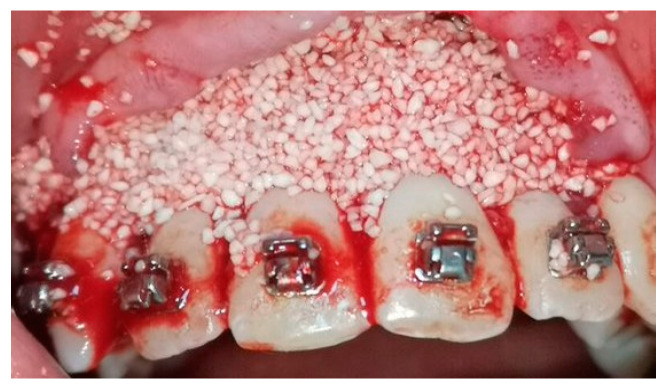
Bone augmentation with mineralized bone.

**Figure 4:**
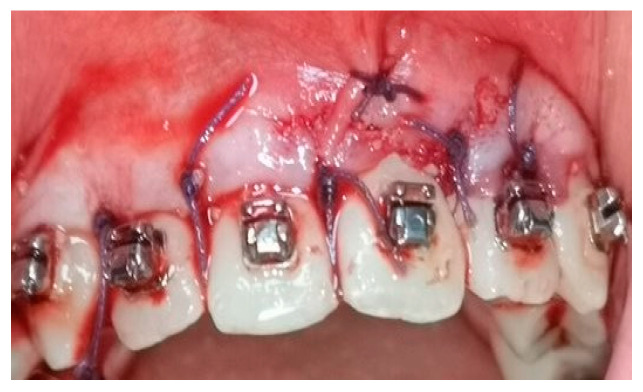
Sutures after the flap reposition.

A crevicular incision on the labial side of the gingiva from the maxillary or mandibular left first premolar to the right first premolar preceded the full-thickness flaps (Figure 1). On the buccal aspect of the cortical bone, a vertical corticotomy was performed with a number 2 round bur under irrigation with saline solution (Figure 2).The length of corticotomy was from 2 to 3 mm below the crest of the alveolar bone to 2 to 3 mm below the apex., followed by bone augmentation using mineralized and deproteinized bone (Bio-Oss, Geistlich AG, Wolhusen, Switzerland) (Figure 3), after which the flap was repositioned and sutured with 6-0 nylon threads (Figure 4). After surgery, a 14 × 25 CUNiTi archwire was inserted, with the activation of the appliance every 2 weeks.

### 2.6. Collection of the Radiological Data

A second CBCT was made 6 months after the corticotomy (T1). The subjects underwent CBCT using a NewTom Vgi Dental Cone Beam Scanner from New Tom, Cefla SC, Bologna, Italy, with a tube voltage of 88 kV and a current intensity of 6 mA, using a cylindrical field of view of 80 mm both in diameter and height. The voxel size was 0.2 mm × 0.2 mm × 0.2 mm. After the measurements were made, the alveolar bone thickness was measured by importing DICOM filess into Mimics software (ver. 18.0; Materalise Mimics Medical, Leuven, Belgium).

Furthermore, in the sagittal view, a line was drawn at the cement enamel junction (CEJ) of the buccal surface. Afterwards, the following radiographic data were collected: the horizontal labial bone thickness measured at the height of 3 mm, 6 mm and 9 mm representing cervical, mid root (medial) and apical points, respectively, and the root length, measured from the CEJ to the apical level. All the radiographic measurements were performed by two orthodontists and a periodontist.

### 2.7. Statistical Analysis

Statistical analysis included elements of descriptive statistics (mean, median and standard deviation) and elements of inferential statistics. The Shapiro–Wilk test was applied to determine the distribution of the analyzed data series. For the comparison of means and medians the Student *t*-test was applied for paired and unpaired data, respectively, using the Wilcoxon test and the Mann–Whitney test. The significance threshold for *p* was 0.05. Statistical analysis was performed using the GraphPad prism trial version, (GraphPad Software, San Diego, CA, USA).

## 3. Results

A total of 60 patients, 32 females (with a mean age range of 39.50 ± 7.87 years) and 28 males (with a mean age range 30.73 ± 6.21 years), were included in this study. Table 1 and Table 2 show the values of the buccal cortical bone thickness measured at T0 and T1 in both females and males, upper and lower arch. For both arches, the most significant changes were observed at the cervical level. In the upper arch, for females at T0 and at T1, 1.36 ± 0.27 mm and 1.82 ± 0.53 mm were measured, respectively. In males, at T0, 1.56 ± 0.36 mm was measured, and at T1, 2.20 ± 0.56 mm. Statistically significant values were obtained at all three levels at which measurements were made.

**Figure 5:**
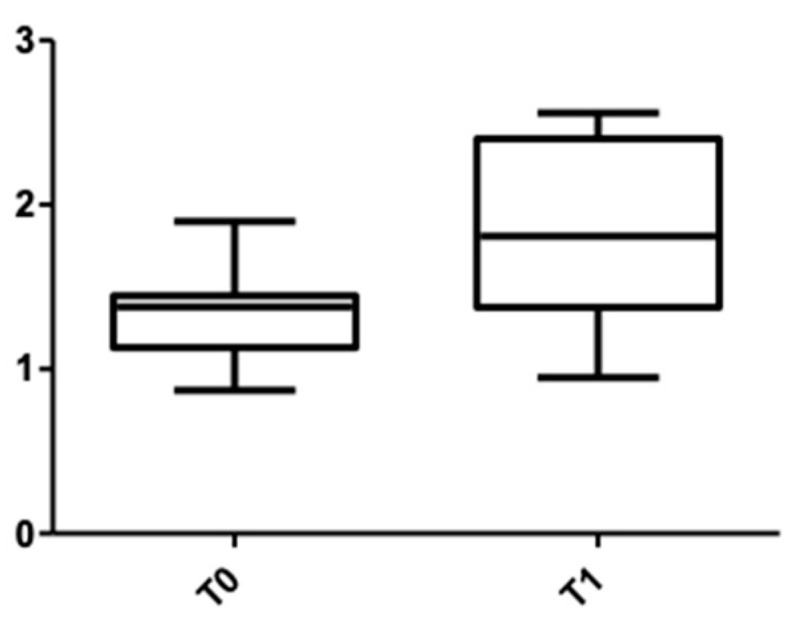
Cone Beam Computer Tomography values at the cervical level, upper arch, at T0 and T1 for females.

**Figure 6:**
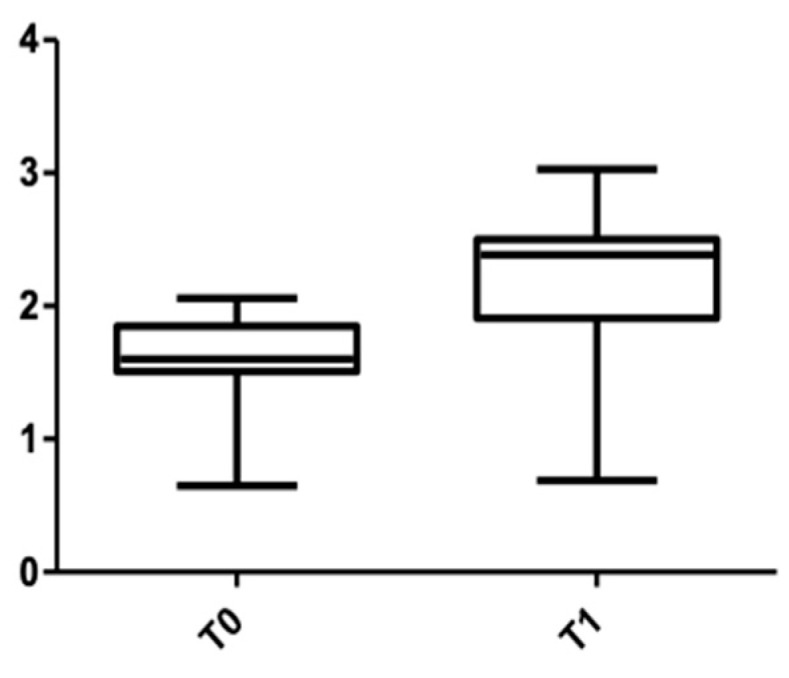
CBCT values at the cervical level, upper arch, at T0 and T1 for males.

Figure 5 and Figure 6 show the distribution of the values obtained for the upper arch at the cervical level for male and female subjects.

**Figure 7:**
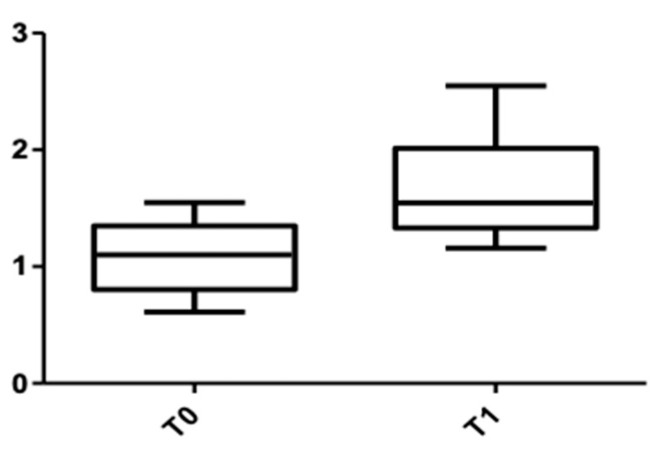
CBCT values at the cervical level, lower arch, at T0 and T1 for females.

**Figure 8:**
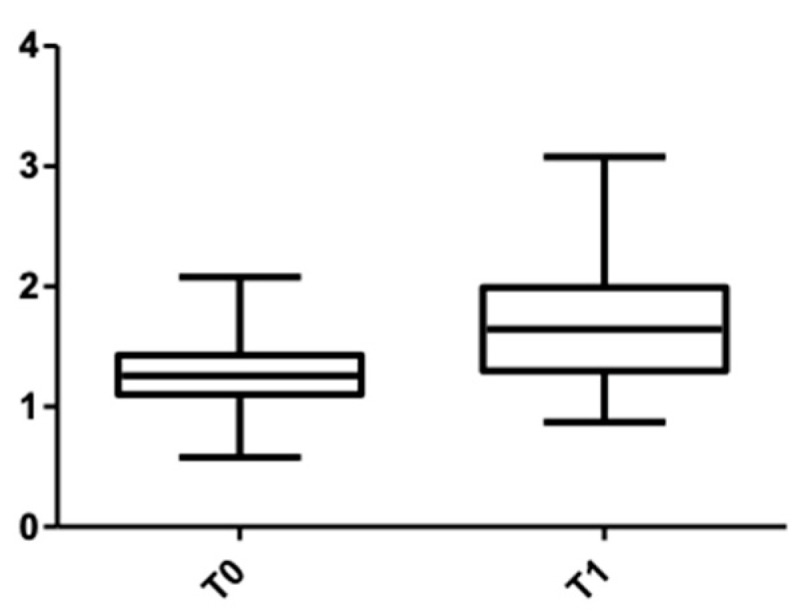
CBCT values at the cervical level, lower arch, at T0 and T1 for males.

Figure 7 and Figure 8 show the distribution of the values obtained when the alveolar mandibular bone thickness was measured at the cervical level.

When individual measurements were made for each tooth (Table 3 and Table 4), it was observed that the thickness of the bone increased at the cervical level, for all teeth included in the study. For the upper right canine, the measured values were 1.49 ± 0.34 mm at T0 and 2.07 ± 0.56 at T1. For the upper left canine, the values obtained on CBCT were 1.47 ± 0.36 mm at T0 and 2.06 ± 0.61 at T1. The lowest values in the upper arch were measured for the second left incisor in the apical area (0.93 ± 0.20 mm at T0 and 1.24 ± 0.36 mm at T1). In the lower arch, the lowest values were observed at the median level, for the left first premolar (0.82 ± 0.16 mm at T0 and 1.11 ± 0.38 mm at T1). The most statistically significant increase in thickness was for the lower right lateral incisor (1.17 ± 0.25 mm at T0 and 1.68 ± 0.43 mm at T1) and lower left central incisor (1.16 ± 0.24 mm at T0 and 1.67 ± 0.39 mm at T1) in the cervical area.

**Figure 9:**
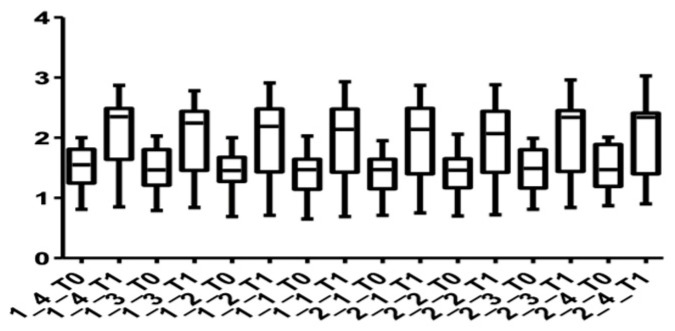
Distribution of CBCT values at the cervical level, upper arch, for all teeth up to the first premolars at T0 and T1.

**Figure 10:**
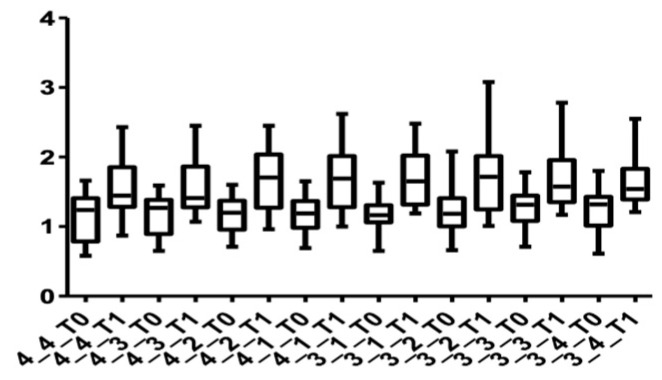
Distribution of CBCT values at the cervical level, lower arch, for all teeth up to the first premolars at T0 and T1.

Figure 9 and Figure 10 show the distribution of the values measured for every group of teeth at the cervical level in both arches.

When the post-treatment root length was measured (Table 5), the value in the upper arch was 11.92 ± 2.24 mm, and it was 13.80 ± 2.28 mm for the lower arch. When the measurements were divided by sex (Table 6), in the maxillary arch, the length measured for males was 14.26 ± 2.06 mm (with 0.2 mm shorter than the initial value), and for females, the root resorption was 0.04 mm (13.38 ± 2.43 mm at T0 and 13.34 ± 2.41 mm at T1). In the mandible, the root resorption was slightly more significant: for female subjects, it was 0.08 mm (12.40 ± 2.07 mm at T0 and 12.32 ± 2.09 mm at T1), and for male subjects, the measured resorption was 0.04 mm (11.57 ± 2.29 mm at T0 and 11.53 ± 2.29 mm at T1).

**Figure 11:**
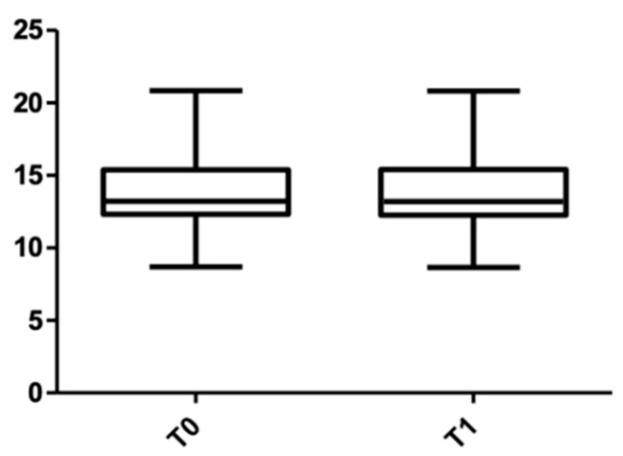
Root length distribution in the upper arch at T0 and T1.

**Figure 12:**
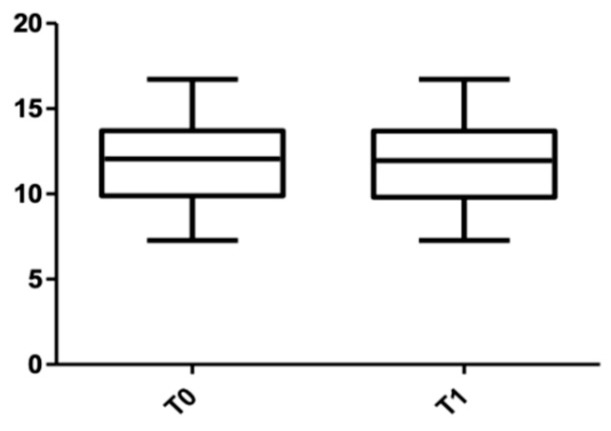
Root length distribution in the lower arch at T0 and T1.

Figure 11 and Figure 12 show the distribution of the root length values at T0 and T1 in both arches.

**Figure 13:**
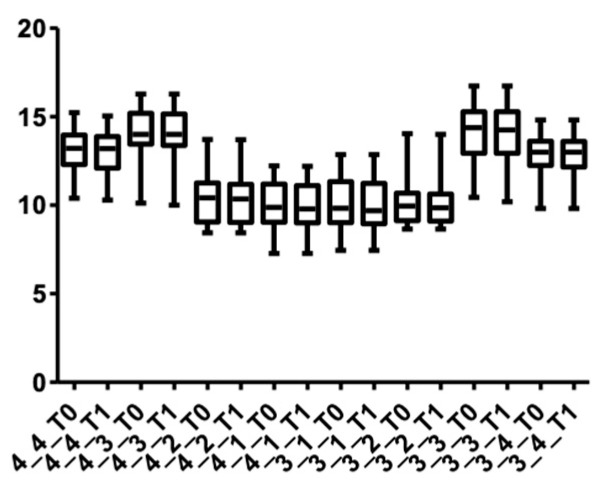
Root length distribution for maxillary teeth at T0 and T1.

**Figure 14:**
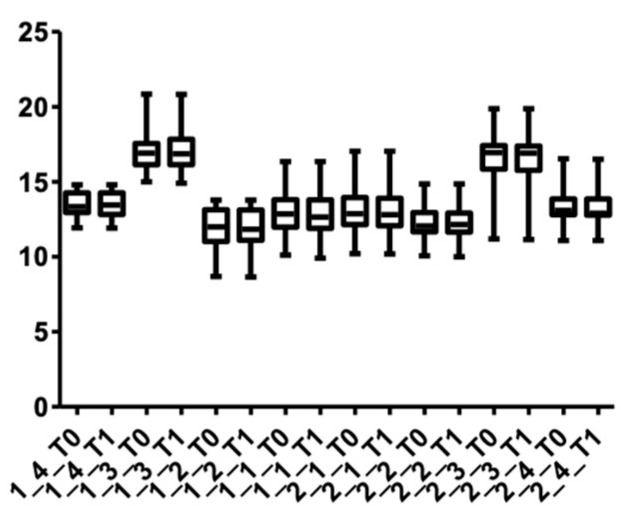
Root length distribution for mandibular teeth at T0 and T1.

With regard to individual measurements of the root length in the upper arch (Table 7), only for the left canine (length was 16.77 ± 1.76 mm at T0 and 16.72 ± 1.78 mm at T1) and the left first premolar (13.34 ± 1.09 mm at T0 and 13.27 ± 1.08 mm at T1) were statistically significant changes present. However, for the right central and lateral incisors, the measured values at T0 and T1 remained unchanged (12.90 ± 1.57 mm and 12.17 ± 1.12 mm, respectively). In the lower arch (Table 8), the most pronounced resorption was measured in the right first premolar (13.05 ± 1.29 mm at T0 and 12.97 ± 1.28 mm at T1). Figure 13 and Figure 14 show the distribution of root length values at T0 and T1 for each tooth.

## 4. Discussion

Dental displacement during orthodontic treatment occurs as a result of resorption and bone apposition [16]. On the other hand, it is well known that following corticotomy, the movements of the dento-alveolar structures appear to be related to the acceleration of the local metabolism, at the level of the area where the intervention is performed [13]. Similar to the healing process of a common fracture, it comprises three phases: the reactive phase, the repair phase and the stage in which the remodeling processes at the bone level takes place [17]. However, it is believed that this acceleration leads to a much faster healing than in the case of physiological repair. The reason for favorable tooth displacement is that the surgery intensifies the activity of osteoblasts and osteoclasts [18]. The intervention consists of a selective decortication of the bone, which leads to a higher rate of transformation of the trabecular bone, together with the appearance of demineralization zones [18,19].

In the present study, following the analysis of the collected data, it was observed that the most significant changes in the buccal bone thickness were registered in the cervical area, in both sexes. On the other hand, the measurements before corticotomy and from a few months after the procedure reveal that the thickness of the buccal cortical bone in the patients included in this study had the greatest thickness in the cervical area, and decreased towards the apical. In a similar study, Ohiomoba et al. concluded that the dimensions of the buccal cortical plates increased as the measurements became more apical with respect to the alveolar ridge [20]. In another study of human skulls, Baumgaertel et al. [21] observed a decrease in the thickness of the cortical plate in the median area, similar to that in our study, where the mandible had a lower cortical thickness in the medial area, compared with the cervical and apical areas.

In a study conducted in 2009 that aimed to analyze the variation in bone density between men and women, Avdagic et al. noticed that boys reached maximum bone density slightly later than girls [22]. This observation supports the result obtained in the present study, where at the mandibular level the measured values in the case of men were lower than those of women, correlated with their younger age compared with the female subjects. On the other hand, various studies claim that women, with age and under the influence of various factors (hormones), have a higher rate of bone loss [23,24]. This may be one of the reasons why, in the present study, the values obtained for the maxillary arch were lower in female subjects, as the average age of the patients was higher.

In a similar study, Seong Ho et al. found that there were no significant differences between the dental groups included in the study; only at the level of the premolars was the thickness of the buccal cortical bone greater [25]. A similar result was obtained in the present study, where the largest thickness was measured at the level of the first premolar in the right maxillary hemiarcade. In the lower arch, higher values were obtained at the level of the incisor group.

This increase in the thickness of the buccal cortical plate could be attributed to both the bone graft material and the RAP phenomenon, which has been observed in such lesions. Our study is consistent with the results obtained in a similar study by Bhattacharya et al., who concluded that orthodontic therapy associated with corticotomy and bone grafting leads to an improvement in alveolar bone support, resulting in an increase in the width of the alveolar process [26].

One of the common side effects that could occur after conventional orthodontic treatment is root resorption. A series of studies in this direction showed that it can begin a few weeks after the initiation of orthodontic treatment but can be observed radiologically only after 3–4 months [27]. This was one of the reasons why in this study, the initial measurements were made after the alignment and leveling of the teeth. This complex biological process occurs when the forces created and applied at the apical level exceed the resilience and adaptability of the tooth [28]. A number of researchers have established a direct correlation between the degree of root resorption and the duration of orthodontic treatment [29,30]. On the other hand, some studies have shown a direct link between corticotomy and the acceleration of tooth displacement, thus implicitly decreasing the duration of treatment [31]. However, in order to support this observation, further studies are needed in this direction, in which several variables are taken into account (the degree of tooth displacement, the direction of force application, the quantity of force, etc.).

Regarding the resorption of various dental groups, most previous studies focused on the incisors of the maxillary arch, as it is assumed that they are the most susceptible to the occurrence of resorption [32,33]. Previous studies have shown that severe resorption occurs in approximately 1% of analyzed teeth, while other dental groups may find varying degrees of resorption [34]. In the present study, when orthodontic therapy was combined with corticotomy, some changes in the root length were observed at the canines and first premolar level in both arches. The same observation was made by Alikhani et al. in a similar study [35].

In many of the studies in which root length was measured, the measurements were taken using panoramic radiographs or periapical radiographs, performed by the parallelization of the long cone. However, when evaluating the degree of root resorption on panoramic radiography, there are a number of shortcomings. Some researchers believe that the values obtained may be overestimated by about 20% [36]. On the other hand, during orthodontic treatment, the angulation of the teeth may change, which would lead to changes in root length visible on radiography [37]. In this study, the determinations were made on the basis of images obtained using CBCT, as the reconstitution is 1:1 and amplification errors are absent. Therefore, studies in which the degree of root resorption was determined using CBCT showed a higher accuracy in the results compared with 2D images [36]. In one study, Wang arrived at the same conclusion, emphasizing the importance of 3D technology when it comes to quantifying root resorption [38].

Over time, various methods have been proposed to enhance the pace of dental displacement, as patients’ reluctance to seek orthodontic treatment is due to its increased duration. One of these methods is the administration of 1.25 dihydroxicolecarciferol and prostaglandin E2. In their study, Kale et al. observed an acceleration of the teeth movement rate, as they claim that these two substances produce some changes in the bone metabolism, in the process of resorption and bone apposition [4]. A similar observation was made in the study by Tyrovola et al., correlating the degree of tooth displacement with the administration of various systemic compounds [5]. In order to shorten the duration of orthodontic treatment, a series of physical stimuli (vibrations) were also applied, which, due to their less invasive nature, could be more easily tolerated by patients compared with other methods. In addition, patients who were given such stimuli reported less discomfort caused by teeth displacement.

In a study performed on an animal model, a higher degree of displacement was observed when the orthodontic force was associated with a vibratory stimulation [39,40]. The application of pulse electromagnetic fields has proven to be another effective way to accelerate tooth movement. When such stimuli were applied to distalize the canine in a post-extractional space, it was observed that on the side where the electromagnetic stimulation was performed, the rate of tooth displacement increased [41].

It is known that the results of clinical trials may vary depending on various parameters, such as particularities related to the geographical area of the subjects involved, and genetic factors [42]. Although there are a number of studies in the literature that highlight the potential benefits of combining corticotomy with orthodontic therapy and changes in dento-periodontal structures, in the literature, at present, there are not enough studies among Romanian patients. 

Although the present study highlights the benefits that may result from corticotomy-assisted orthodontics, there are some limitations, namely, the number of subjects included in the study, as well as the short period of time during which the evaluation was performed. A larger number of subjects, but also the type of malocclusion that was treated, could lead to different results. Therefore, further research is needed to confirm the results obtained in our study.

## 5. Conclusions

- It has been observed that when combined with conventional orthodontic treatment, corticotomy can lead to an increase in the bone thickness supporting the tooth; changes observed in the buccal cortical plate support this observation.
- Although changes were observed in all three levels at which the measurements were made, the area where the corticotomy has the most significant effect is the cervical.
- In the maxillary arch, the most significant changes were registered at the level of the canines and first premolars, and in the lower arch at the incisors level.
- The bone support reacted differently to the stimuli that appeared after corticotomy, depending on gender, in correlation with age and local metabolism. Therefore, changes in the cortex were greater in male subjects when the average age of female subjects was high, knowing that bone metabolism in women decreases with age and under the influence of hormones.
- Root resorption, a side effect associated with conventional orthodontic therapy, was found to be much diminished, with statistically insignificant differential values.

## Figures and Tables

**Table 1:** Comparison of the level of buccal bone thickness at T0 and T1 in both sexes in the maxillary group, based on Cone Beam Computer Tomography values.

Sex	CBCT Values Upper Arch	T0 Median ± SD (mm)	T1 Median ± SD (mm)	T1−T0 (mm)	*p*-Value
Females	Cervical	1.363 ± 0.276	1.829 ± 0.539	0.466	<0.0001
	Medial	1.090 ± 0.120	1.468 ± 0.427	0.377	<0.0001
	Apical	0.920 ± 0.171	1.281 ± 0.434	0.361	<0.0001
Males	Cervical	1.561 ± 0.365	2.201 ± 0.568	0.640	<0.0001
	Medial	1.095 ± 0.261	1.626 ± 0.405	0.531	<0.0001
	Apical	1.000 ± 0.207	1.304 ± 0.342	0.304	<0.0001

**Table 2:** Comparison of the level of buccal bone thickness at T0 and T1 in both sexes in the mandibular group.

Sex	CBCT Values Lower Arch	T0 Median ± SD (mm)	T1 Median ± SD (mm)	T1−T0 (mm)	*p*-Value
Females	Cervical	1.097 ± 0.276	1.684 ± 0.420	0.586	<0.0001
	Medial	0.747 ± 0.197	1.073 ± 0.500	0.325	<0.0001
	Apical	1.125 ± 0.307	1.561 ± 0.437	0.436	<0.0001
Males	Cervical	1.260 ± 0.273	1.640 ± 0.422	0.379	<0.0001
	Medial	0.818 ± 0.158	1.120 ± 0.315	0.301	<0.0001
	Apical	1.321 ± 0.297	1.692 ± 0.392	0.370	<0.0001

**Table 3:** Comparison of the level of buccal bone thickness at T0 and T1 for the maxillary teeth.

Tooth	CBCT Values Upper Arch	T0 Median ± SD (mm)	T1 Median ± SD (mm)	T1−T0 (mm)	*p*-Value
14	Cervical	1.524 ± 0.337	2.101 ± 0.568	0.577	<0.0001
	Medial	1.151 ± 0.186	1.627 ± 0.448	0.475	<0.0001
	Apical	1.011 ± 0.170	1.349 ± 0.396	0.338	0.0004
13	Cervical	1.491 ± 0.342	2.072 ± 0.567	0.581	0.0001
	Medial	1.116 ± 0.179	1.586 ± 0.423	0.469	0.0001
	Apical	1.007 ± 0.166	1.377 ± 0.403	0.370	0.0002
12	Cervical	1.448 ± 0.336	1.993 ± 0.589	0.545	<0.0001
	Medial	1.096 ± 0.195	1.517 ± 0.393	0.420	<0.0001
	Apical	0.969 ± 0.172	1.290 ± 0.363	0.320	0.0003
11	Cervical	1.426 ± 0.345	1.971 ± 0.607	0.545	<0.0001
	Medial	1.074 ± 0.211	1.510 ± 0.397	0.436	0.0001
	Apical	0.933 ± 0.204	1.256 ± 0.402	0.322	0.0006
21	Cervical	1.413 ± 0.316	1.977 ± 0.603	0.563	<0.0001
	Medial	1.077 ± 0.218	1.477 ± 0.394	0.400	0.0001
	Apical	0.929 ± 0.213	1.254 ± 0.385	0.325	0.0004
22	Cervical	1.415 ± 0.341	1.951 ± 0.598	0.536	<0.0001
	Medial	1.060 ± 0.222	1.445 ± 0.382	0.384	0.0002
	Apical	0.931 ± 0.203	1.245 ± 0.366	0.313	0.0005
23	Cervical	1.479 ± 0.364	2.064 ± 0.615	0.584	0.0002
	Medial	1.074 ± 0.214	1.596 ± 0.456	0.521	<0.0001
	Apical	0.947 ± 0.183	1.337 ± 0.423	0.389	0.0004
24	Cervical	1.501 ± 0.360	2.075 ± 0.603	0.574	<0.0001
	Medial	1.093 ± 0.213	1.618 ± 0.507	0.525	0.0001
	Apical	0.954 ± 0.240	1.313 ± 0.424	0.359	0.0003

**Table 4:** Comparison of the level of buccal bone thickness at T0 and T1 for the mandibular teeth.

Tooth	CBCT Values Lower Arch	T0 Median ± SD (mm)	T1 Median ± SD (mm)	T1−T0 (mm)	*p*-Value
44	Cervical	1.154 ± 0.318	1.568 ± 0.405	0.414	0.0002
	Medial	0.715 ± 0.187	1.085 ± 0.397	0.313	0.0016
	Apical	1.288 ± 0.309	1.644 ± 0.426	0.356	0.0002
43	Cervical	1.190 ± 0.267	1.588 ± 0.405	0.398	0.0003
	Medial	0.803 ± 0.198	1.118 ± 0.397	0.314	0.0007
	Apical	1.248 ± 0.308	1.617 ± 0.451	0.369	0.0003
42	Cervical	1.171 ± 0.256	1.686 ± 0.438	0.515	<0.0001
	Medial	0.765 ± 0.165	1.077 ± 0.363	0.311	0.0007
	Apical	1.205 ± 0.307	1.622 ± 0.397	0.417	0.0001
41	Cervical	1.176 ± 0.275	1.683 ± 0.452	0.507	<0.0001
	Medial	0.816 ± 0.169	1.118 ± 0.463	0.301	0.0005
	Apical	1.212 ± 0.294	1.640 ± 0.381	0.428	<0.0001
31	Cervical	1.165 ± 0.244	1.676 ± 0.394	0.515	0.0002
	Medial	0.765 ± 0.188	1.094 ± 0.409	0.329	0.0005
	Apical	1.187 ± 0.321	1.619 ± 0.388	0.432	0.0001
32	Cervical	1.210 ± 0.337	1.718 ± 0.528	0.507	<0.0001
	Medial	0.796 ± 0.206	1.117 ± 0.426	0.320	0.0007
	Apical	1.237 ± 0.332	1.654 ± 0.468	0.417	<0.0001
33	Cervical	1.280 ± 0.276	1.696 ± 0.427	0.416	0.0003
	Medial	0.778 ± 0.157	1.088 ± 0.406	0.310	0.0005
	Apical	1.283 ± 0.309	1.648 ± 0.373	0.365	0.0002
34	Cervical	1.215 ± 0.322	1.645 ± 0.340	0.430	0.0003
	Medial	0.823 ± 0.164	1.113 ± 0.388	0.290	0.0013
	Apical	1.286 ± 0.376	1.674 ± 0.483	0.387	0.0003

**Table 5:** Root length at T0 and T1 for maxillary and mandibular teeth.

Root Length	T0 Median ± SD (mm)	T1 Median ± SD (mm)	*p*-Value
Lower arch	11.981 ± 2.242	11.972 ± 2.244	0.210
Upper arch	13.830 ± 2.283	13.811 ± 2.285	0.187

**Table 6:** Root length at T0 and T1 for maxillary and mandibular teeth in female and male patients.

Root Length	Sex	T0 Median ± SD (mm)	T1 Median ± SD (mm)	*p*-Value
Lower arch	Females	12.401 ± 2.078	12.380 ± 2.098	0.216
	Males	11.572 ± 2.296	11.562 ± 2.292	0.183
Upper arch	Females	13.381 ± 2.434	13.365 ± 2.415	0.260
	Males	14.283 ± 2.037	14.264 ± 2.064	0.118

**Table 7:** Root length at T0 and T1 for maxillary teeth.

Root Length	T0 Median ± SD (mm)	T1 Median ± SD (mm)	*p*-Value
14	13.456 ± 0.816	13.432 ± 0.833	0.674
13	17.042 ± 1.422	17.020 ± 1.470	0.363
12	11.890 ± 1.324	11.864 ± 1.330	0.329
11	12.903 ± 1.531	12.906 ± 1.577	0.945
21	13.072 ± 1.524	13.043 ± 1.600	0.509
22	12.174 ± 1.189	12.174 ± 1.121	0.832
23	16.771 ± 1.762	16.722 ± 1.785	0.002
24	13.341 ± 1.096	13.271 ± 1.086	0.002

**Table 8:** Root length at T0 and T1 for mandibular teeth.

Root Length	T0 Median ± SD (mm)	T1 Median ± SD (mm)	*p*-Value
44	13.05 ± 1.298	13.00 ± 1.286	0.003
43	14.16 ± 1.481	14.10 ± 1.495	0.024
42	10.49 ± 1.595	10.48 ± 1.593	0.210
41	10.05 ± 1.320	10.04 ± 1.313	0.309
31	10.10 ± 1.489	10.10 ± 1.488	0.180
32	10.32 ± 1.550	10.32 ± 1.560	0.435
33	14.20 ± 1.664	14.15 ± 1.699	0.008
34	12.84 ± 1.216	12.79 ± 1.234	0.043

## Data Availability

The data presented in this study are available on request from the corresponding author. The data are not publicly available due to patient confidentiality.
